# ATR-FTIR Analysis and One-Week Stress Relaxation of Four Orthodontic Aligner Materials

**DOI:** 10.3390/ma13081868

**Published:** 2020-04-16

**Authors:** Florina Jaggy, Spiros Zinelis, Georgios Polychronis, Raphael Patcas, Marc Schätzle, George Eliades, Theodore Eliades

**Affiliations:** 1 Clinic of Orthodontics and Pediatric Dentistry, Center of Dental Medicine, University of Zurich, 8032 Zurich, Switzerland; florina.jaggy@uzh.ch (F.J.); raphael.patcas@zzm.uzh.ch (R.P.); schaema@hotmail.com (M.S.); 2 Department of Biomaterials, School of Dentistry, National and Kapodistrian University of Athens, 11527 Athens, Greece; szinelis@dent.uoa.gr (S.Z.); gpolyg@yahoo.com (G.P.); geliad@dent.uoa.gr (G.E.)

**Keywords:** orthodontic aligners, relaxation, FTIR, optical microscopy, PETG, shear band

## Abstract

The aim of this study was to estimate possible differences in the chemical composition and relaxation of orthodontic aligner materials. Four commercially available thermoplastic materials CAM (Scheu-Dental, Iserlohn, Germany), COP (Essix, Dentsply Raintree Essix Sarasota, FL, USA), DUR (Great Lakes Dental Technologies, Tonawanda, NY) and ERK (Erkodent Erich Kopp, Pfalzgrafenweiler Germany) were included in this study. Rectangular strips from each material were prepared according to the manufacturer’s instructions and subjected to attenuated total reflection-Fourier transform infrared (ATR-FTIR) spectroscopy and stress relaxation characterization. The reduction in applied stress (RAS) after one week was estimated and statistically analyzed by one-way ANOVA at the 0.05 level of significance. All specimens were subjected to optical microscopy before and after stress relaxation testing under transmittance polarized illumination. ATR-FTIR microscopy revealed that all materials are made of polyethylene terephthalate glycol (PETG) while no significant differences were identified in RAS values among materials tested, which ranged from 6%–10% (p ≥ 0.05). All samples illustrated the developments of shear bands during relaxation testing according to optical microscopy findings. The tested materials illustrated similar chemical composition and relaxation behavior and thus no differences in their clinical efficacy are anticipated.

## 1. Introduction

The introduction of Invisalign system at the end of 1990s by Align Technology (San Jose, California, USA) was a breakthrough for orthodontic aesthetic treatment with removable appliances. The fabricated sequential positioners were based on polyurethane [1] and had the ability to move teeth in small increments without causing major patient discomfort [2] nor undermining their oral hygiene [3,4,5,6,7]. The majority of manufacturers have changed the initially utilized thermoplastic material with polyethylene terephthalate glycol (PETG) and fabricate aligners of variable thicknesses. Regardless of the chemical compositions of the raw material used, the biomechanics [8,9,10] and the mode of action behind aligners remain similar. Specifically, every positioner is subject to deformation upon intraoral placement, and the initial magnitude of force placed on the teeth depends both on material stiffness and thickness as well as the intended tooth displacement prescription [11,12]. Upholding the magnitude of force over the applied time, which usually does not exceed 1 to 2 weeks, is a crucial aspect for a predictable orthodontic tooth movement. Hence force relaxation, defined as the reduction of force over time at standard deformation, is of great importance when considering aligners and their potential of deformation. In contrast to superelastic Ni-Ti wires, which can be placed for longer periods of time intraorally without significant effects on their mechanical properties, the thermoplastic materials are viscoelastic in nature [13] and consequently are prone to stress relaxation. The relevance of this characteristic in regard to the efficacy of orthodontic treatment is self-evident. Previous in vitro studies have confirmed the rapid decrease of force magnitude as a result of stress relaxation [13,14,15,16]. However, in many cases stress relaxation curves did not exhibit a definite plateau phase. This might be attributed to the limited amount of stress relaxation tests and their short duration ranging from a few hours up to a day. Thus, the clinical relevance of the previous studies may be challenged, as most manufacturers recommend the usage of each aligner for at least one week and the added time may further compromise the clinical performance.

The aim of this research was to determine the in vitro stress relaxation phenomenon and the associated changes of chemical and structural properties of aligners from different brands for a period of time of one week. The null hypothesis of this study was that no statistical difference exists in chemical composition and mechanical properties among the studied materials.

## 2. Materials and Methods 

### 2.1. Materials

Four commercially available thermoplastic materials for the production of orthodontic aligners were included in this study. The brand names, the manufacturers and the codes of all materials are presented in Table 1. For sample preparation five thermoplastic sheets from each material were used. The thermoplastic sheets were pressed over a rectangular dental stone model employing the manufacturers’ instructions.

### 2.2. ATR-FTIR Spectroscopy

One strip from each material was cut by a lancet and spectra were acquired by attenuated total reflectance Fourier transform infrared (ATR-FTIR) spectroscopy. The samples were placed against the diamond reflective elements of a single reflection ATR accessory equipped with SnSe lenses (Golden Gate, Specac Inc., Smyrna, GA, USA) and pressed with a sapphire anvil to achieve firm contact with the diamond crystal. Spectra were obtained using an FTIR spectrometer (Spectrum GX, Perkin-Elmer Corp, Bacon, UK) under the following acquisition parameters: range 4000-650 cm^−1^, resolution 4 cm^−1^ and 20 scans co-addition. All spectra were subjected to ATR and baseline corrections while Peak fitting analysis was performed by PeakFit v.4.12 software (Seasolve, Framingham, MA, USA). All experiments were replicated in triplicate. 

To study the ratio of free (C=O) versus hydrogen bonded (C=O…H) carboxyl groups the peak of the 1650–1750 cm^−1^ wavenumber range were curve fitted employing a Gaussian algorithm (peak area mode) at standard width/variable shape mode and 2% zero baseline. The net area ratio (Ri) of hydrogen bonded carboxyl groups (C=O…H; 1710) to free ones (C=O; 1728 cm^−1^) were calculated as an index of intermolecular bonding among materials tested.

### 2.3. Relaxation

One rectangular specimen of 30 × 4 × (0.3~0.5) mm in dimension was cut from each pressed model employing a dental lancet. The dimensions of each sample were measured with a digital caliper (Mitutoyo, Tokyo, Japan). The rectangular samples were placed in a custom-made unit consisting of a 2-Kg load cell (RS components 632-736, RDP Electronics, Wolverhampton, UK) connected with a signal conditioning unit (E307-3 RDP Electronics). The latter was linked to an ADC-16 (Analog to Digital Converter) multichannel data acquisition unit and the signal was transmitted to a PC equipped with a data logging software (Picolog technology systems, Cambridgeshire, UK) providing data logging of force over time. Then an initial force which corresponds to 2.1MPa was applied and force over time was recorded once per minute for a total period of one week (168 hours). The rational for the selection of this initial stress is thoroughly explained in the discussion section. The final stress after one week was calculated and the reduction in applied stress (RAS) after one week was expressed in percentage according to the formula:

RAS(%)=100% (S_1week_ – S_initial_)/S_initial_ (1)

### 2.4. Optical Microscopy

All samples were imaged before and after relaxation testing in an optical microscopy (DM 4000B/ Leica Microsystems, Wetzlar, Germany) under transmittance polarized illumination and representative images were acquired from different locations under 50×, 100× and 200× nominal magnifications.

### 2.5. Statistical Analysis

The results of RAS were initially checked for normality and homoscedacity by Kolmogorov– Smirnov and equal variance test respectively and then were statistically analyzed employing a one-way Analysis of variance (a = 0.05).

## 3. Results

### 3.1. ATR-FTIR Spectroscopy

**Figure 1:**
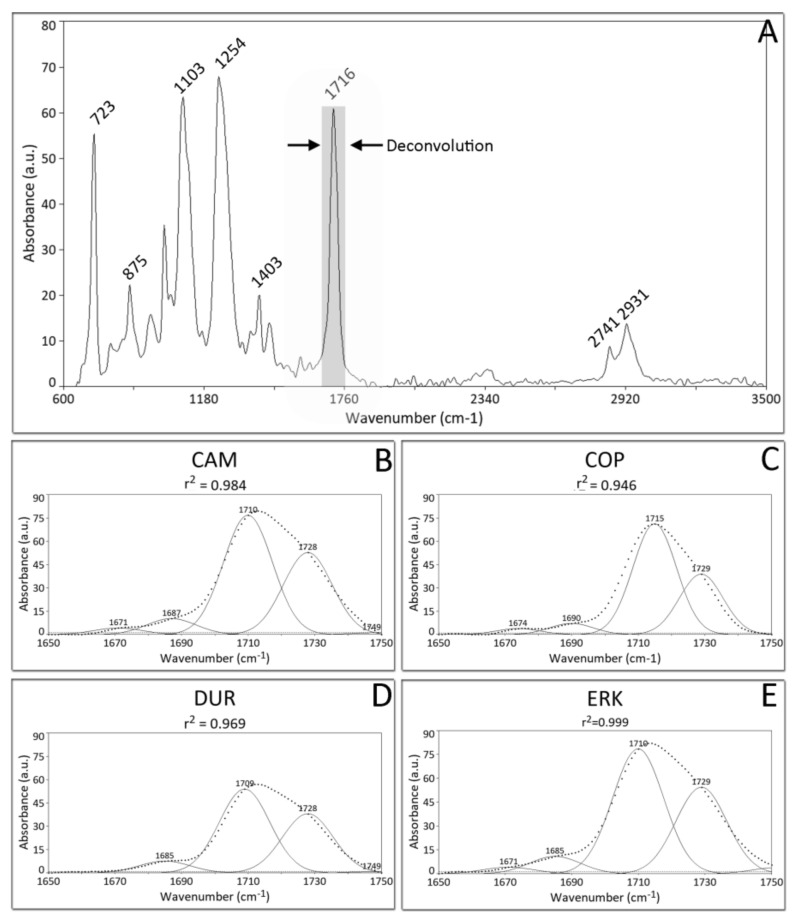
(**A**) Representative ATR-FTIR spectrum from all material tested. (**B**–**E**) Gaussian curve fitted spectra of carboxyl peak at (C=O, 1716 cm^−1^) for CAM, COP, DUR and ERK respectively.

All materials tested showed identical ATR-FTIR spectra and Figure 1 (A) shows a representative spectrum which includes the characteristic bands of CH (2931, 2741, 1403, 723 cm^-1^), C=O (1716 cm^−1^), aromatic CH (875, 1505 cm^−1^) and C-C-O-(1254, 1103 cm^−1^). Figure 1B–E show the deconvolution of the main carboxyl peak at 1716 of CAM, COP, DUR and COP respectively. The index Ri classified the tested materials in the following descending order: (Ri): COP = 1.63, DUR = 1.46, ERK = 1.45, CAM = 1.41.

### 3.2. Relaxation

**Figure 2:**
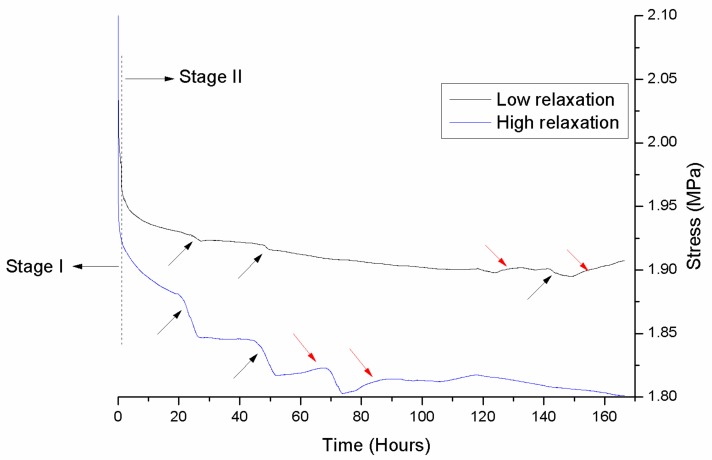
Representative stress–time curves from all materials tested over an observation time of 7 days. High and low relaxation curves were found for all materials. All curves are characterized by a first stage of an abrupt decrease of stress (Stage I), lasting approximately 2 hours, and a second stage (Stage II) with a low decrease of stress. Both curves demonstrate areas with sudden decrease of stress (black arrows) which in most cases are followed by a small increase of stress (red arrows).

**Figure 3:**
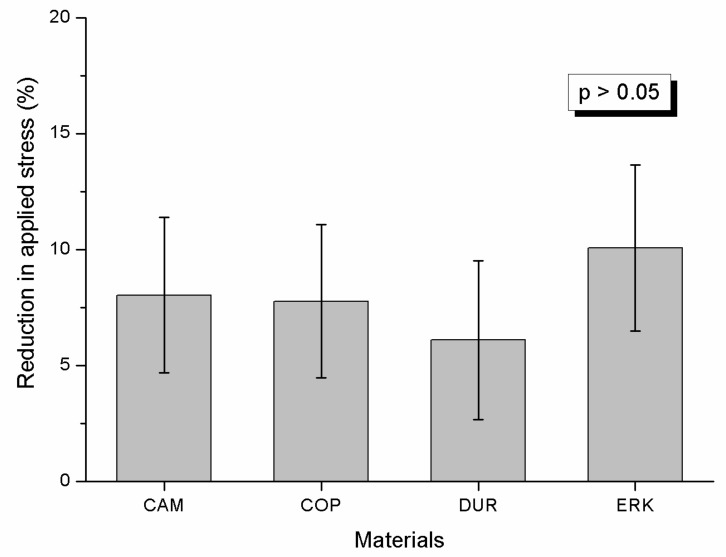
Box and whiskers plots of mean values and standard deviations of reduction in applied stress after 1 week for all materials tested. No statistically significant differences were identified (*p* > 0.05).

Figure 2 illustrates representative stress–time curves. The recorded curves were not identical and demonstrated low and high relaxation. However, for all curves a short first stage (Stage I) of approximately 2 hours defined by a high rate decrease of stress was noticeable, which was followed by a second stage with a low rate decrease of stress up to the end of the recording time (7 days). Interestingly, all curves demonstrated in Stage II points with a sudden decrease of stress (pointed by black arrows) which in most cases were followed by periods with a slight increase in stress. This was common in both low and high relaxation curves. The results of RAS are presented in Figure 3. Statistical analysis disclosed that no significant differences were detected among the tested materials (*p* > 0.05).

### 3.3. Optical Microscopy

**Figure 4:**
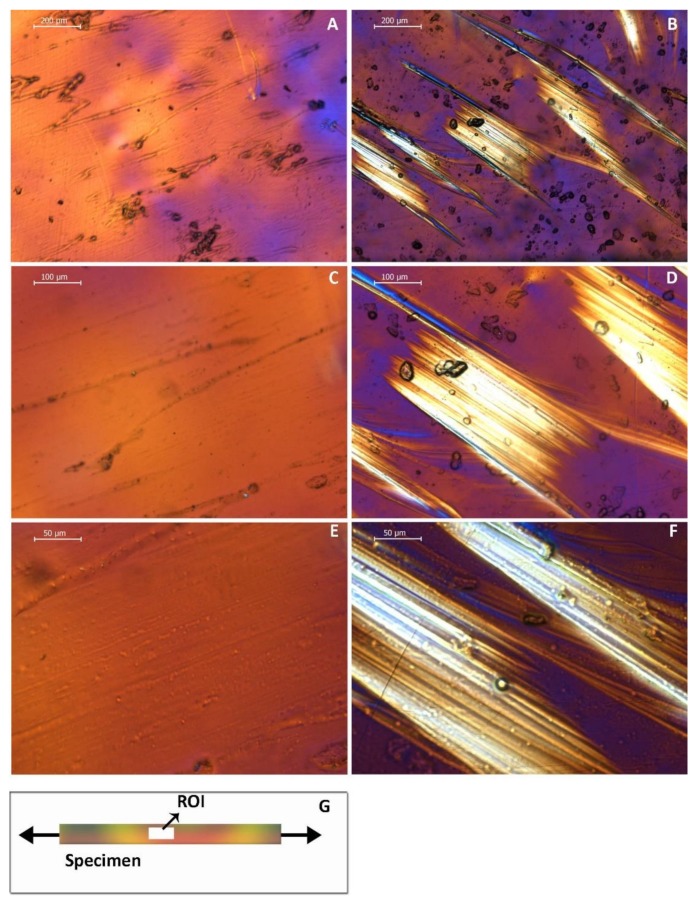
Optical microscope images under transmittance polarized illumination from a sample before (**A**,**C**,**E**) and after relaxation testing (**B**,**D**,**F**) at different magnifications. (**G**) The image shows that the horizontal axes of photographs are parallel to the long axis of the sample. After relaxation the sample reveals the development of bright regions with an orientation approximately 45^o^ to horizontal axis. The images were taken with original magnifications of 50× (**A**,**B**), 100× (**C**,**D**) and 200× (**E**,**F**).

Figure 4 demonstrates images under transmittance polarized illumination from a sample before and after relaxation testing, at 50×, 100× and 200× nominal magnification. The horizontal axis of all images is parallel to the long axis of the sample. The development of bright regions is easily identified in the sample treated for relaxation (Figure 1B,D,E). These regions demonstrate an orientation with a 45^o^ angle to the long axis of the sample.

## 4. Discussion

Statistically significant differences could be substantiated, neither for chemical composition nor for the mechanical properties tested and thus the null hypothesis could not be rejected.

All materials showed identical ATR-FTIR spectra which match that of polyethylene glycol terephthalate (PETG) [17], a material that has been already described for the manufacturing of orthodontic aligners in dental literature [1,18,19,20]. Despite the similarity of the materials tested, the Ri index (the ratio of hydrogen bonded carboxyl groups to free ones) shows that COP has a higher intermolecular bonding than CAM, followed by ERK and DUR, implying that different productions of the raw material and/or dissimilar manufacturing processes of orthodontic aligners may impact the extent of intermolecular structure of orthodontic aligners. 

Although the multifaceted milieu of intraoral ageing cannot be simulated in experimental studies adequately, it is important to formulate testing conditions that are similar at least to the clinical setting. Previous studies [13,14] dealing with the stress relaxation of orthodontic aligners have used very high initial stresses (up to 40 MPa, a value close to fracture strength of PETG [19]) and shorter duration (up to 24 h), which are unrealistic and irrelevant simulations. Based on in vivo data with strain gauges analysis, it has been estimated that orthodontic aligners undergo a maximum strain of 3500 microstrain [21], which is equal to 0.0035 strain. Two previous studies employing tensile testing have estimated the modulus of elasticity of PETG between 450 [19] and 750 MPa [22] and thus the average of 600 MPa was used to calculate the applied stress based on the fundamental equation that σ = E * ε where σ, E and ε stand for stress, modulus of elasticity and strain, respectively. Therefore, based on the dimension of each specimen the required force was applied, providing for all specimens a standard initial force of 2.1 MPa. Despite the very low initial stress applied, all materials demonstrated relaxation, validating the results of previous experimental studies [13,14,23] and recently published investigations that relaxation index is decreased after intraoral aging of one week compared to control material [24,25]. The results of this present study suggest that relaxation of orthodontic aligners should be considered as a degradation mechanism during intraoral conditions. At the same time, it demonstrates the necessity to introduce alternative raw materials in the form of shape memory polymers, with decreased relaxation potential, which would also be less prone to hydrolytic degradation.

No significant differences were identified (Figure 3) for the reduction of applied stress after one week, implying that all materials undergo equal degradation despite their differences in Ri. The latter denotes that the higher intermolecular bonding does not provide better relaxation resistance for the material tested, and possible advancements to increase this property should be researched in alternative mechanisms. The optical microscopy analysis (Figure 4) showed that all samples developed new bright regions with an orientation approximately 45^o^ to the long axis of the specimens and thus may be characterized as shear bands, a known behavior of PETG material under tensile loading [26]. In general, under tensile loading the vector of maximum shear develops at an angle of 45^o^ and 135^o^ to tensile vector and this facilitates the development of shear bands with such an orientation. A shear band (commonly known as “strain localization”) is determined as a region of intense shearing strain indicating an abrupt loss of homogeneity of material. The nucleation and development of shear bands may be associated with the abrupt decrease (pointed by black arrows) or slight increase of stress (red arrows) during Stage II of relaxation curves (Figure 2) but this is only an assumption and requires further experimental testing. 

From a clinical point of view, the results of this study indicate that the orthodontic aligners lose a substantial amount of their initial force during the very first hours, and continue to steadily decrease their force over the following days. Clinicians should be made cognizant that aligners do not have the capacity to exert a steady magnitude of force over a prolonged time, and that the loss of force seems to be best characterized by a two-stage process, unrelated to the brand used.

Although the applied initial stress can be considered close to intraoral conditions based on the aforementioned analysis, the results of this study cannot be directly extrapolated to clinical practice as the effect of softening mechanism due to wet oral environmental has been completely omitted, which remains an obvious limitation of this study. Given that previous studies have shown that the water has a profound effect on stress relaxation behavior of thermoplastic aligners [13], the relaxation under intraoral conditions is anticipated to be even higher than the values portrayed in the current study.

## 5. Conclusions

The commercially available material tested share similar relaxation behavior and chemical composition.

The differences in intermolecular bonding do not affect the relaxation behavior.

## Figures and Tables

**Table 1:** Brand names, manufactures, and codes for all materials tested.

Brand Name	Company	Code
CA-medium	Scheu-Dental, Iserlohn, Germany	CAM
Copolyester	Essix, Dentsply Raintree Essix Sarasota, FL, USA	COP
Duran	Great Lakes Dental Technologies, Tonawanda, NY	DUR
Erkodur	Erkodent Erich Kopp, Pfalzgrafenweiler Germany	ERK
